# Understanding the Vibrational Structure and Ultrafast Dynamics of the Metal Carbonyl Precatalyst [Mn(ppy)(CO)_4_]

**DOI:** 10.1021/acsphyschemau.4c00037

**Published:** 2024-07-09

**Authors:** Jonathan B. Eastwood, Barbara Procacci, Sabina Gurung, Jason M. Lynam, Neil T. Hunt

**Affiliations:** † Department of Chemistry and York Biomedical Research Institute, University of York, York YO10 5DD, U.K.; ‡ Department of Chemistry, University of York, York YO10 5DD, U.K.

**Keywords:** ultrafast spectroscopy, 2D-IR spectroscopy, homogeneous catalysis, manganese-catalyzed C−H bond activation, solvent−solute interaction

## Abstract

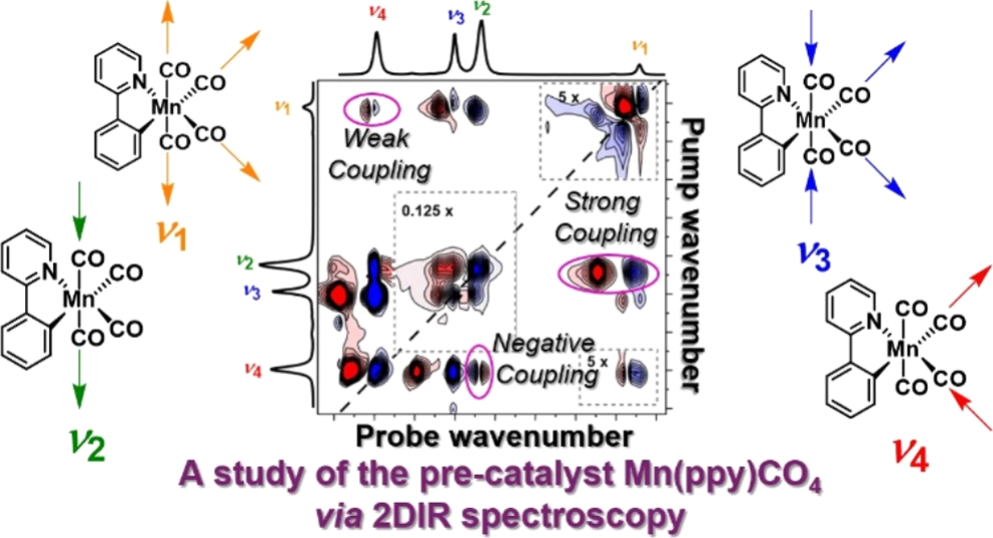

The solution phase structure, vibrational spectroscopy, and ultrafast relaxation dynamics of the precatalyst species [Mn(ppy)(CO)_4_] (**1**) in solution have been investigated using ultrafast two-dimensional infrared (2D-IR) spectroscopy. By comparing 2D-IR data with the results of anharmonic density functional theory (DFT) calculations, we establish an excellent agreement between measured and predicted inter-mode couplings of the carbonyl stretching vibrational modes of **1** that relates to the atomic displacements of axial and equatorial ligands in the modes and the nature of the molecular orbitals involved in M–CO bonding. Measurements of IR pump–probe spectra and 2D-IR spectra as a function of waiting time reveal the presence of ultrafast (few ps) intramolecular vibrational energy redistribution between carbonyl stretching modes prior to vibrational relaxation. The vibrational relaxation times of the CO-stretching modes of **1** are found to be relatively solvent-insensitive, suggestive of limited solvent–solute interactions in the ground electronic state. Overall, these data provide a detailed picture of the complex potential energy surface, bonding and vibrational dynamics of **1**, establishing a fundamental basis for the next steps in understanding and modulating precatalyst behavior.

## Introduction

Transition-metal carbonyl complexes play a vital role as catalysts in the synthesis of bulk and fine chemicals, while their commercial use as catalysts for hydroformylation^1^ and acetic acid formation through methanol carbonylation is well documented.^2^ Much of the interest in using carbon monoxide as a ligand in transition-metal organometallic chemistry arises from the fact that it is an industrially available C_1_ synthon that binds strongly to electron-rich, low oxidation state d-block metal complexes. The latter property is a primary consequence of the well-established σ-donor and π-acceptor interactions between metal- and CO-based orbitals. In an octahedral complex, the metal d-orbitals with *T*_2*g*_ symmetry become strongly bonding, ensuring that transition-metal carbonyl compounds have a strong ligand field. This contrasts with the general case whereby 3d-metal complexes show weaker metal–ligand interactions^3^ and so exhibit smaller ligand fields than their 4d and 5d counterparts, making them more prone to one-electron oxidation reactions.^4^ The tendency for CO ligands in 3d-metal complexes to enforce low-spin electronic configurations and reduce unwanted redox reactions^5^ has thus led to significant interest in their development as sustainable alternatives to the currently used 4d and 5d equivalents.^4,6−8^

A second important consequence of the σ-donor and π-acceptor interactions between metals and carbonyl ligands is that the extent of the (mainly) π-acceptor interaction modulates the M–C and C≡O bond strengths, which can be interrogated through changes to the frequencies of the vibrational modes of the carbonyl ligands.^9^ Coupled with the strong dipole moment of the C≡O bond, this means that infrared (IR) spectroscopy is an ideal tool to investigate the structure and dynamics of transition-metal carbonyl compounds.

**Figure 1:**
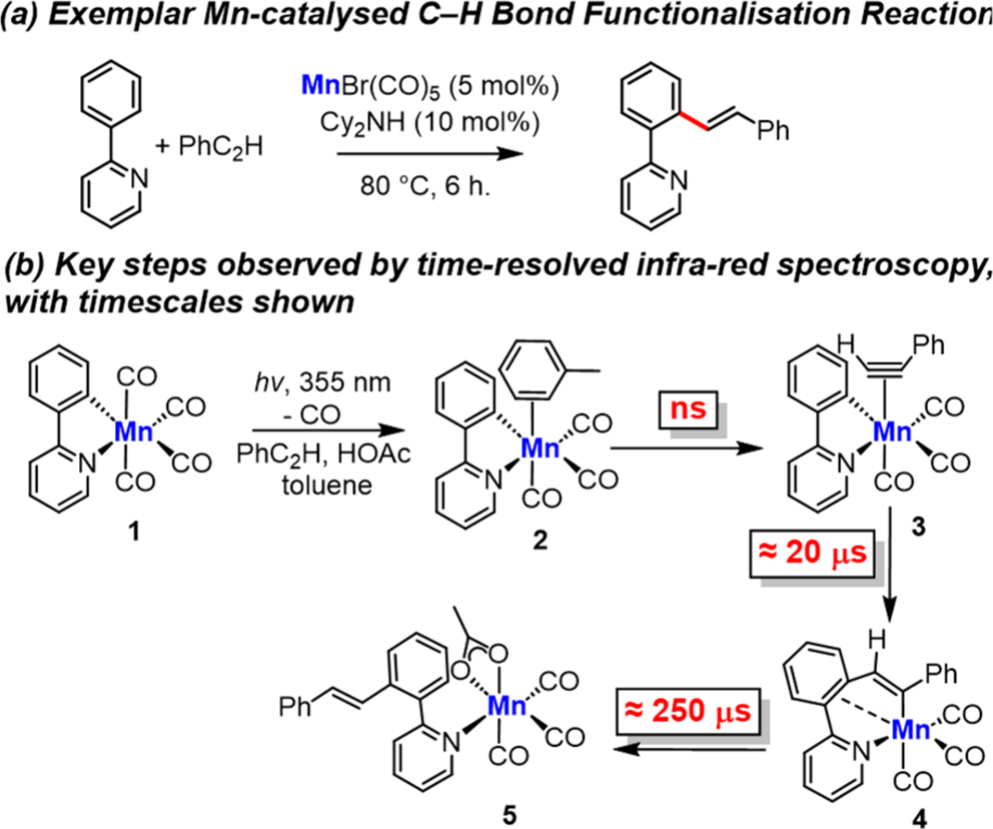
(a) Exemplar Mn-catalyzed C–H bond functionalization reaction^10^ and (b) summary of results from previous time-resolved spectroscopic studies involving **1**.^5^

The combination of 3d-metal carbonyls and IR spectroscopic analysis, including ultrafast time-resolved methods, has been used successfully in the application of simple manganese carbonyl compounds, such as [MnBr(CO)_5_] and [Mn_2_(CO)_10_], to C–H bond functionalization reactions that couple heterocyclic substrates with a range of different electrophiles (Figure 1a). Detailed mechanistic studies have shown that cyclomanganated compounds, such as [Mn(ppy)(CO)_4_],^7,10−12^**1** (ppy = cyclometalated 2-phenylpyridine) are key intermediates and viable precatalysts in these reactions. The key catalytic steps mediated by **1** have been investigated by time-resolved ultraviolet (UV) pump–IR probe spectroscopy experiments spanning time scales from ps to ms (Figure 1b). In these experiments, the UV pump induces an ultrafast CO dissociation from **1**,^13^ simulating the same process that happens under thermal catalytic conditions. This results in the formation of complexes *fac*-[Mn(ppy)(CO)_3_S] (S = solvent molecule) and insight into the nature of the photoproducts was obtained from the energy and symmetry of the strong vibrational modes of the three remaining CO ligands. By observing changes to the bands over time, details of the subsequent dynamics of the complex were obtained. In complex mixtures that simulate catalytic reactions, it was further demonstrated that the initial binding event is kinetically controlled^14,15^ with Mn coordinating in the first place to the solvent (the most abundant species) to form **2** (in toluene solution) (Figure 1b). A ligand substitution by, for example, phenylacetylene then yielded **3**. Importantly, it was possible to directly observe the migratory insertion reaction of the alkyne into the Mn–C bond, a key step underpinning the catalytic reaction, to give **4**.^16−19^ Performing the experiment in the presence of HOAc allowed for the observation of a slower protonolysis of the Mn–C bond to reveal **5**,^20^ which has the product from the reaction bound to the metal. Therefore, this approach has enabled key catalytic steps mediated by the metal to be directly observed.^5^

The ability of IR spectral analysis of the photoproducts formed from **1** to give unique insights into the mechanistic behavior of the Mn precatalyst establishes a requirement to obtain a comprehensive understanding of the nature of the vibrational modes of this molecule. Such information will not only support the assignment of time-resolved spectra but also provide a means to interpret the fundamental nature of the precatalyst and its interaction with the molecular environment to guide future modifications. Of particular importance is the question of the vibrational coupling of the CO-stretching modes (ν_CO_) to one another and with the organic ligand, as changing the structure of this group profoundly influences reactivity.^14,15,18^ A related issue is the mechanism of vibrational energy management by the complex, which will impact reaction dynamics.

In this manuscript, we report an investigation into the carbonyl ligand vibrational manifold of [Mn(ppy)(CO)_4_] (Figure 1b, **1**) using ultrafast 2D-IR spectroscopy. 2D-IR has been widely applied to study metallocarbonyl species, providing insights into vibrational potential energy surfaces,^21−23^ solvent–solute interactions,^24−29^ equilibrium dynamics,^30^ and real-time evolution of photoactivated molecules.^24,31−34^ These have included several studies of catalytically relevant species^26,31−33^ including bioinspired systems.^35−38^ By comparing 2D-IR data with anharmonic density functional theory (DFT) calculations, we establish inter-mode couplings and solution phase structural parameters for the ν_CO_ modes of **1** in addition to measuring the vibrational relaxation dynamics of **1** in a range of solvents relevant to catalytic functionality. We show that these data provide a detailed picture of the complex potential energy surfaces and bonding of **1**, establishing a fundamental basis for the next steps in understanding precatalyst behavior.

## Experimental Section

### Synthesis and Characterization of **1**

Compound **1** was synthesized and characterized following procedures reported elsewhere.^39^ A detailed description is provided in the electronic Supporting Information (ESI).

### Sample Preparation for IR Spectroscopy

The samples for all IR spectroscopy experiments were prepared by dissolving 0.8 mg/mL **1** in dry deoxygenated *n*-heptane solvent (∼2.5 mM). A 500 μL aliquot of this solution was placed into a transmission cell (Harrick) featuring two CaF_2_ windows separated by a PTFE spacer (thickness: 200 μm) to specify the optical path length.

### IR Absorption Spectroscopy

IR absorption spectra were recorded in transmission mode at room temperature using a Bruker Vertex 80 FTIR spectrometer with a spectral resolution of 1 cm^–1^. The empty spectrometer under the N_2_ atmosphere was used to acquire background spectra for the measurements. All spectra reported were the average of 32 scans.

### 2D-IR Spectroscopy

The two-dimensional infrared (2D-IR) spectrometer consisted of two Yb-based amplified lasers (Pharos 20W and Pharos 10W, Light Conversion) synchronized by a single, common oscillator. The amplifiers were each used to pump an optical parametric amplifier (OPA, Orpheus Mid-IR, Light Conversion) equipped with difference frequency generation (Lyra, Light Conversion) to produce independently tunable sources for one- or two-color 2D-IR spectroscopy. In the mid-IR, the two OPAs produce usable bandwidths of >200 cm^–1^ with energies of 2.5 and 1.5 μJ/pulse, respectively, at a pulse repetition rate of 50 kHz.

2D-IR data collection was achieved using a 2DQuick spectrometer (Phasetech). The spectrometer employs the pump–probe beam geometry for 2D-IR data collection^40−42^ and uses a mid-IR pulse shaper to generate and control the time delay (τ) between the pair of “pump” pulses. The waiting time (*T*_w_) between the second pump and probe pulses was determined by an optical delay line. Signal measurement was achieved via twin 64-element HgCdTe array detectors configured to allow simultaneous collection of either signal and reference or ZZZZ (parallel) and ZZYY (perpendicular) polarization-resolved data.

In the experiments that follow, the output of OPA pumped by the Pharos 20W amplifier was used to create all three infrared pulses (two pumps, one probe). All pulses were centered at 2000 cm^–1^, resonant with the ν_CO_ modes of **1**. For a given value of *T*_w_, τ was scanned in steps of 20 fs to a maximum delay time of 4 ps applying a rotating frame frequency of 1585 cm^–1^. Each 2D-IR plot represents the average of 500 spectra, repeated 3 times, taking ∼2 min to acquire.

### Density Functional Theory Calculations

DFT calculations were performed with Gaussian 16, Revision C.02,^43^ using the bp86 functional and def2-sv(p) basis set and an ultrafine integration grid. The heptane solvent was treated with an SCRF model. The geometry of **1** was optimized and confirmed as a minimum by an absence of negative frequency vibrational modes. Anharmonic vibrational frequency calculations were performed using the *freq* = *anharm* keyword.

## Results

### Infrared Absorption Spectroscopy

**Figure 2:**
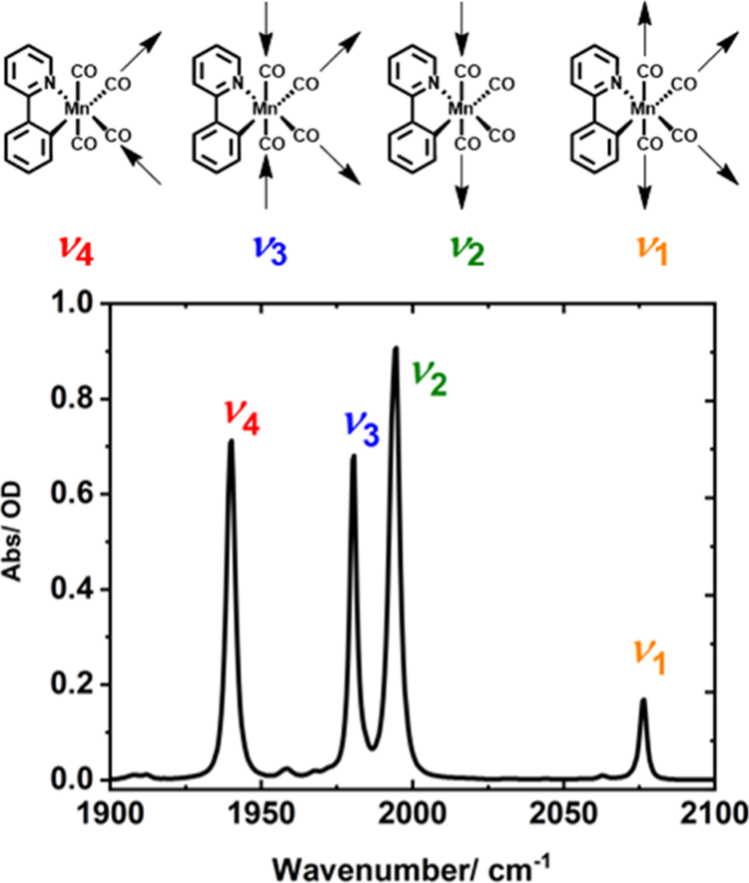
Top: Schematic diagram illustrating the vibrational modes of [Mn(ppy)(CO)_4_] **1**. Bottom: FTIR spectrum of 0.8 mg/mL solution of **1** in *n*-heptane.

The IR spectrum of **1** in heptane solution is shown in Figure 2. Four bands are visible in the region of 1940–2080 cm^–1^, consistent with previous studies.^13^ These bands have been assigned to the ν_CO_ modes (ν_1–4_) shown in Figure 2. Based on a *C*_*s*_ point group description of **1**, we identify these as 3*A*′(ν_1,3,4_) and 1*A*″ (ν_2_) symmetry modes. To aid with a comparison with the related isoelectronic systems [Cr(bpy)(CO)_4_]^44^ and [Re(bpy)(CO)_4_]^+^^45^ (bpy = 2,2′-bipyridyl), we note that if an approximation to the higher symmetry *C*_2*v*_ point group for **1** is assumed, then these can be assigned to 2*A*_1_ (ν_1,3_), *B*_1_ (ν_2_), and *B*_2_ (ν_4_). For clarity, in the discussion that follows, we will refer to the bands using the ν_1–4_ terminology shown in Figure 2.

### 2D-IR Spectroscopy

**Figure 3:**
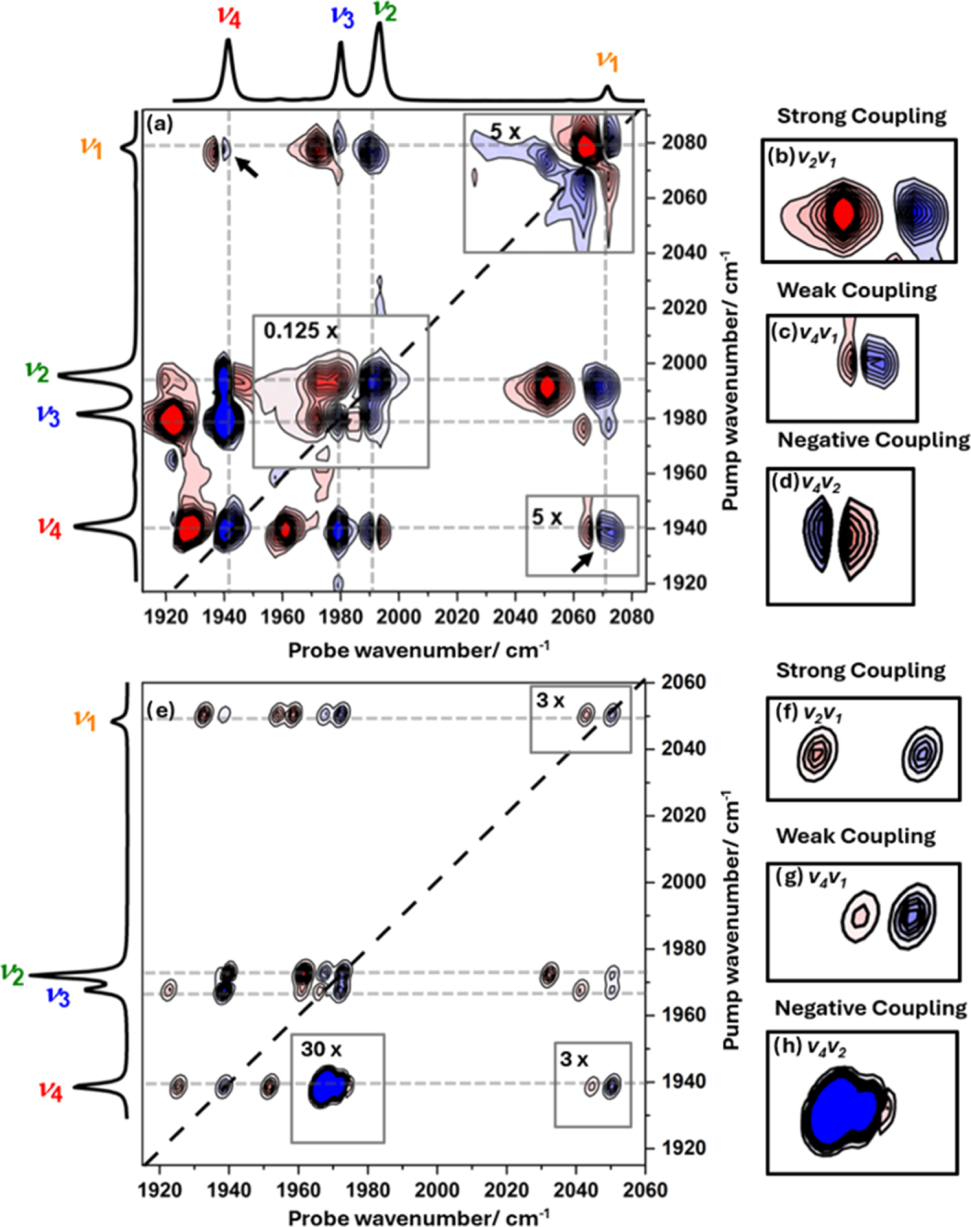
(a) 2D-IR spectrum of **1** in *n*-heptane solution measured at *T*_w_ = 500 fs using ZZYY polarization alongside IR absorption spectra for comparison. Regions of the 2D-IR spectrum have been scaled for clarity and these are indicated by gray boxes with the magnification factor shown. (b–d) Enlarged sections of the spectrum in (a) focusing on off-diagonal peaks, which exemplify (b) strong coupling between modes ν_2_ and ν_1_, (c) weak coupling between modes ν_4_ and ν_1_, and (d) negative coupling between modes ν_4_ and ν_2_ as discussed in the text. Panel (e) shows a simulated 2D-IR spectrum based on the results of anharmonic DFT calculations for comparison (see the text and SI). (f–h) The same expanded regions in the predicted spectrum as in (b–d). The color scale for all plots runs from blue (negative) to red (positive).

The 2D-IR spectrum of **1** in heptane solution at a *T*_w_ value of 500 fs (Figure 3a) shows a number of peaks. On the diagonal of the spectrum (pump frequency = probe frequency, dashed black line), four blue (negative) peaks can be seen, which coincide with the four bands in the IR absorption spectrum of **1**, which is shown alongside the 2D-IR plot for comparison. These diagonal peaks can be assigned to the v = 0–1 transitions of the ν_1–4_ modes. Each blue diagonal peak is accompanied by a red (positive) peak shifted between 8 and 12 cm^–1^ to lower probe frequency. These peaks are assigned to the corresponding v = 1–2 transitions of the ν_1–4_ modes. The frequency separation of the positive and negative (v = 1–2 and v = 0–1) peaks is due to the anharmonicity of the vibrational potentials. By fitting Gaussian lineshapes to horizontal slices through the spectrum in Figure 3a at pump frequencies corresponding to modes ν_1_ to ν_4_, the values of the anharmonic shifts for each of the four modes were evaluated and these are listed in Table 1 (left side, gray boxes).

**Table 1:**
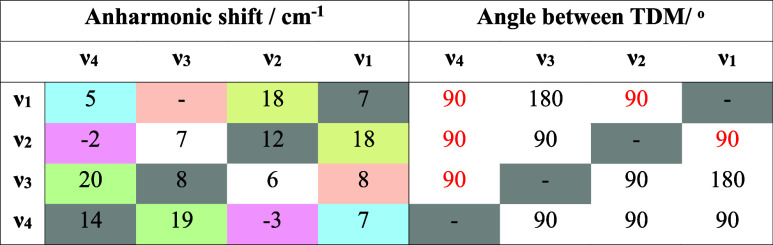
Left: Diagonal and Off-diagonal Anharmonicities Derived from Figure 3a (cm^–1^)^a^

a Gray cells represent the diagonal anharmonicities, and colored cells highlight off-diagonal anharmonicities between common pairs of modes. The pumped mode is indicated in the left-hand column so that the layout of the table resembles the 2D-IR spectrum. Right: Angles (°) between transition dipole moments (TDM) of modes ν_1–4_ determined from 2D-IR data by off-diagonal peak anisotropy values (see the text). Figures in red show experimentally determined values while those in black are predicted via DFT calculations. Note the agreement between measured and predicted values for the same pairs of modes appearing on the opposite sides of the diagonal, which is indicated by gray boxes.

Also visible in Figure 3a are peaks in the off-diagonal part of the 2D-IR spectrum. At short values of *T*_w_ relative to the vibrational relaxation time of **1** (see below), as in Figure 3a (*T*_w_ = 500 fs), these off-diagonal peaks indicate the presence of vibrational coupling between the ν_CO_ modes. Using the gray dashed guidelines (Figure 3a) it is clear that each of the four diagonal peaks is linked to the remaining three modes by such off-diagonal features, which each appear as a pair of negative and positive peaks. These peak pairs are most clearly visible in the top left and bottom right corners of the spectrum in Figure 3a, identified with arrows in Figure 3a. The off-diagonal peaks marked with arrows can be assigned to the coupling of modes ν_1_ to ν_4_ (ν_1_ ν_4_; top left, where the first term of ν_1_ ν_4_ indicates the pumped mode) and the reverse process (ν_4_ ν_1_; bottom right), respectively. The spectroscopic origin of the off-diagonal peak pair can be understood using the ν_1_ ν_4_ off-diagonal feature as an example. The pumped mode is ν_1_ and the presence of negative and positive components in the off-diagonal feature arises from transitions due to the v = 0–1 transition of the coupled mode (ν_4_, negative) and a transition (positive peak) from v = 1 of the pumped mode (ν_1_) to the combination band featuring one quantum of excitation in both of the modes (one each in ν_1_ and ν_4_). The vibrational coupling of modes ν_1_ and ν_4_ causes the energy of the combination state to be shifted in energy from the sum of the two fundamental transitions by the off-diagonal anharmonicity, which is a direct measure of the coupling strength of the two modes. This means that, for any pair of coupled modes, we can obtain a measure of the coupling strength from the separation of the negative and positive components of the off-diagonal peak linking the respective diagonal peaks.^42^

Once again, fitting Gaussian lineshapes to horizontal slices through the spectrum allows these off-diagonal anharmonic shifts, or coupling strengths, to be evaluated for each pair of ν_CO_ modes of **1** (Table 1, colored cells). The values vary quite markedly from the very strong (ν_1_ν_2_ and ν_3_ν_4_: 18–20 cm^–1^), exemplified in Figure 3b, to comparatively weak (ν_1_ν_4_, ν_2_ν_3_, and ν_3_ν_1_: 5–8 cm^–1^, Figure 3c). One particularly interesting example shows the presence of negative coupling between modes ν_2_ and ν_4_ (ν_2_ν_4_ = −2 cm^–1^), for which the probe frequency ordering of the negative and positive components of the off-diagonal peak pair is reversed relative to all of the others (Figure 3d).

In addition to obtaining coupling parameters between the pairs of ν_CO_ modes, the off-diagonal peaks in a 2D-IR spectrum can be used to extract information relating to the angles between transition dipole moment (TDM) directions for each pair of modes.^22,42^ This is achieved by comparing the relative amplitudes of diagonal and off-diagonal peaks linking a given pair of coupled vibrational modes in 2D-IR spectra obtained using parallel (ZZZZ) and perpendicular (ZZYY) pulse polarization geometries.^22,42^ Performing this analysis for off-diagonal peaks that are relatively free of strong overlaps with other peaks allowed extraction of angles of 90°, within experimental error, for ν_1_ν_2_, ν_1_ν_4_, ν_2_ν_4_, and ν_3_ν_4_ (red figures in Table 1 and Figure S1).

### DFT Calculations

Anharmonic DFT calculations were used to predict the vibrational frequencies and intensities of the fundamental ν_CO_ transitions of **1** as well as the frequencies of transitions from the v = 1 levels of each mode to higher-lying vibrational states. As this enables predictions of the v = 1–2 transition frequencies for each of the ν_CO_ modes and the frequencies of transitions to combination states, direct comparisons of the results of the calculation with all of the transition frequencies extracted from the 2D-IR spectroscopy data are possible. The numerical results of the simulations are shown in Table S1, but for ease of comparison with the 2D-IR data, we have used the values to produce a simulated 2D-IR spectrum (Figure 3e) in which peak positions produced from the DFT-predicted transitions are represented by a 2D-Gaussian lineshape function (see the SI). The result of this process shows that the agreement between experiment and theory is excellent. Although the precise predictions of the (unscaled) frequencies of the fundamental bands are slightly different from the measured values, as would be anticipated, the mode separations are well reproduced along with the relative intensities of the bands in the IR absorption spectrum. Of particular relevance to our 2D-IR study, there is very close agreement between the coupling parameters obtained from experiment and calculation. This is manifest in the accurate reproduction of the experimental off-diagonal peak patterns (Figure 3a) in the simulated spectrum (Figure 3e) including those with strong, weak, and negative couplings measured for **1**, highlighted above (see Figure 3f–h).

**Figure 4:**
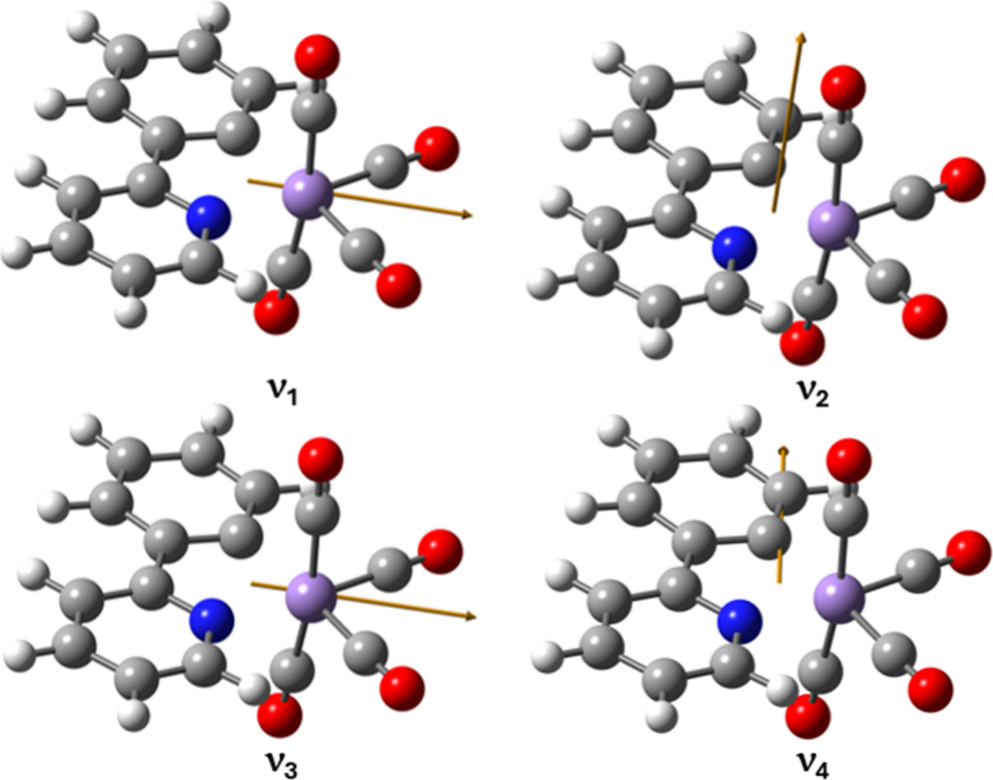
Figure showing the vectors of the transition dipole moments associated with each of the ν_CO_ modes of **1**.

The outputs of the DFT calculations were also used to predict the vectors of TDMs for each of the ν_CO_ modes (Figure 4), and the angles between them agree very well with those calculated from the polarization-resolved 2D-IR spectra (*cf* red and black values in Table 1). This not only provides confidence in the band assignments of the modes in the spectrum but also confirms the solution phase structure of the precatalyst for the first time.

### Vibrational Dynamics

**Figure 5:**
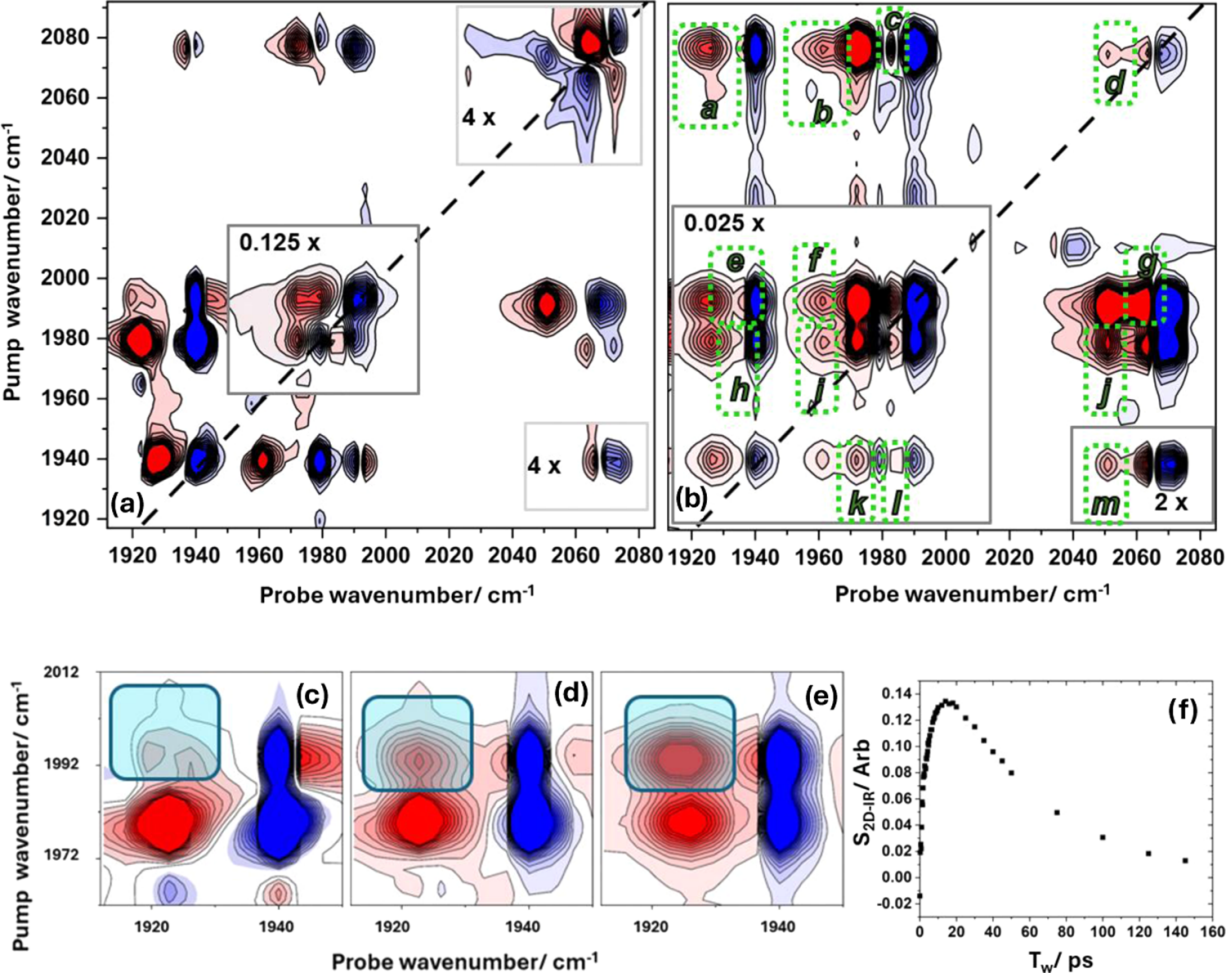
(a) 2D-IR spectrum of **1** in *n*-heptane solution measured at *T*_w_ of 500 fs (ZZYY). (b) 2D-IR spectrum of **1** in *n*-heptane solution measured at *T*_w_ of 20 ps (ZZYY). New peaks appearing due to energy transfer (IVR) are highlighted using green boxes and labeled (a–m). The spectrum in (b) has been scaled by a factor of 40 to make smaller peaks visible. Panels (c–f): Expanded regions of 2D-IR spectra of **1** at a number of values of *T*_w_ showing the temporal evolution of peak e from (b). *T*_W_ values are (c) 250 fs, (d) 2.0 ps, and (e) 14 ps. The off-diagonal peak e, highlighted by the box, increases in intensity due to IVR. The full temporal evolution of the peak is shown in (f), where the amplitude decay is due to vibrational relaxation. In each of the 2D-IR plots, the color scale runs from red (positive) to blue (negative). The scale is common to all plots.

Alongside the IR spectroscopy and solution phase structure of **1** in the electronic ground state, 2D-IR, in combination with IR pump–probe spectroscopy, was used to interrogate the vibrational energy relaxation mechanisms and rotational dynamics of **1** in solution. The effect of vibrational relaxation on the 2D-IR spectrum of **1** is shown in Figure 5 (and over a larger range of time scales in Figure S2) in which the spectrum at a short value of *T*_w_ (500 fs) is compared with one obtained at *T*_w_ = 20 ps. Three main changes to the spectrum occur during the increased *T*_w_ period: (1) All of the peaks in the spectrum diminish in absolute intensity; (2) off-diagonal peaks observed in the short *T*_w_ spectrum are still present, but somewhat larger relative to the diagonal peak with the same pump frequency; and (3) a set of new peaks appear in the spectrum, and these are highlighted by green boxes in Figure 5b and labeled (a–m).

#### Vibrational Relaxation

The overall reduction in peak amplitudes across the spectrum as *T*_w_ increases occurs as a result of vibrational relaxation. The pump process in a 2D-IR experiment leads to a population of the v = 1 levels of modes ν_1_–ν_4_. These populations relax to the respective ground vibrational states with dynamics governed by the vibrational lifetime (*T*_1_) of the v = 1 level of each of the modes. For simplicity, the *T*_1_ time scales for each of the modes of **1** were determined using IR pump–probe spectroscopy, and results from a number of solvents were compared. The recovered vibrational relaxation time for each ν_CO_ mode (Figure S3) is shown in Table 2. It is noticeable from these results that, despite the solvents ranging from nonpolar heptane and toluene to polar THF and acetonitrile, the vibrational relaxation time does not show the kind of large variations that are often observed for metallocarbonyls in solution,^28,34,46^ with the values in all four solvents falling in the range of 30–50 ps. Indeed, the values of 44–59 ps for modes ν_1_–ν_4_ determined for **1** in heptane are considerably faster than might be expected based on previous studies of alkane-solvated metallocarbonyls.^46−48^

**Table 2:** Vibrational Lifetimes (v_1_–v_4_, ps) and Anisotropy Parameters (v_2_, ps) Determined by IR Pump–Probe Spectroscopy for **1** in Solvents of Different Properties^a^

	vibrational relaxation, τ/ps				anisotropy ν_2_
solvent	ν_1_	ν_2_	ν_3_	ν_4_	τ/ps
*n*-heptane	59 ± 5	48 ± 1	44 ± 2	49 ± 1	13 ± 3
toluene	38 ± 1	36 ± 1		38 ± 1	7 ± 1
THF	37 ± 2	31 ± 2		36 ± 1	8 ± 2
CH_3_CN	44 ± 1	40 ± 2		42 ± 1	4 ± 1

a The experimental data were best represented by a biexponential decay function including a fast component of ∼1 ps for both the vibrational relaxation and the anisotropy, which is assigned to an instrument response.

#### Intramolecular Vibrational Redistribution

The growth of off-diagonal peak amplitudes relative to the diagonal peaks and the appearance of new peaks in the 2D-IR spectrum as *T*_w_ increases have been observed in similar systems previously^22^ and can both be assigned to the process of intramolecular vibrational redistribution (IVR). In this process, the pump pulse excites a given mode (ν_1_–ν_4_) of **1**, populating the v = 1 level, but the fast transfer of this population between all of the ν_CO_ v = 1 levels occurs so that, on the IVR time scale, an equilibrium is established between the v = 1 populations. This population then relaxes with the *T*_1_ times of the individual modes.

While IVR is often manifest in the dynamics of diagonal and off-diagonal peaks via the appearance of biexponential behavior, the clearest indication of IVR in the 2D-IR spectrum of **1** can be seen via the appearance of new peaks in the spectrum as *T*_w_ increases (Figure 5b). These are observed because population transfer from the v = 1 level of the pumped mode to the v = 1 level of a nonpumped mode allows observation of the v = 1–2 transition of the newly populated mode, which is not present at shorter values of *T*_w_. This leads to new, positive off-diagonal peaks appearing at the v = 1–2 frequency of the nonpumped mode. In a system such as **1**, where strong mode couplings mean that the positions of the positive components of the off-diagonal peaks caused by coupling (visible at short *T*_w_, Figure 5a) are separated from the positions of the v = 1–2 transitions of the modes populated by IVR by more than the spectral line width, new peaks appear. This is further exemplified in Figure 5c–f and highlighted by the cyan boxes and labeled (a–m).

Measuring 2D-IR spectra as a function of *T*_w_ (Figure S2) and fitting the growth time scales of the new bands due to IVR (a–m) to exponential functions (Figure S4) enabled quantification of the IVR time scales for each of the new peaks (Table 3). While mode dependent, IVR generally occurs on sub-10 ps time scales for **1** in heptane, though the two examples where the pumped and IVR-populated mode show the greatest frequency separation (peaks a and m), involving energy transfer between modes ν_1_ and ν_4_, show the longest time scales (∼13–20 ps). This is as expected because conservation of energy requires the mismatch in energies between the pumped and IVR-populated modes to be made up via the low-frequency modes of the solvent acting as a thermal bath for receiving or donating energy.^49−51^ The latter modes are populated according to the Boltzmann distribution and represent a reservoir of up to ∼200 cm^–1^ at room temperature. In the case of modes ν_1_ and ν_4_, which are separated by 140 cm^–1^, IVR is occurring through the tail of this thermal distribution leading to a reduced rate.^52^ It is noticeable that the downhill energy transfer rate, where the pumped mode lies higher in energy than the receiving mode (peak a; ν_1_–ν_4_, τ = 13 ps), is faster than the uphill process (peak m, ν_4_–ν_1_, τ = 22 ps), consistent with the principle of detailed balance.

**Table 3:** IVR Time Scales (ps) for the Growth of Energy Transfer Peaks a–m^a^

IVR peak	τ ps	approx mode separation cm^–1^
a	13 ± 2	140
b		100
c	5 ± 1	85
d	2 ± 1	
e	2 ± 1	55
f	4 ± 1	15
g	3 ± 1	85
h	8 ± 2	40
i		
j	5 ± 1	100
k	6 ± 1	40
l	11 ± 2	55
m	22 ± 3	140

a The nomenclature refers to the assignments given in Figure 5b. Mode separations are identified using pairs of diagonal modes (ν_1–4_) that link the new off-diagonal features appearing due to IVR.

#### Anisotropy

The results of IR pump–probe spectroscopy measurements obtained as a function of input pulse polarization were used to investigate the rotational dynamics of **1** in solution via the anisotropy parameter (Figure S5 and Table 2). Focusing on the ν_2_ mode of **1**, which yielded the strongest signals, the anisotropy was found to be well represented by a double exponential function in all solvents. The shorter component was found to be less than 1 ps and is assigned to an instrument response. The longer component in heptane was 13 ps, which we assign to rotational relaxation of **1**. This is consistent with rotational time scales observed for metallocarbonyls of similar size in alkane solutions.^26,38^ In more polar solvents, the observed time scale reduced to 4–8 ps. However, the separation between this value and the measured IVR time scale for **1** makes it difficult to assign the time scale definitively to molecular rotation because IVR between ν_CO_ modes will lead to a scrambling of the dipole moment direction of the molecules, similar to the effect of molecular reorientation.

#### Coherent Dynamics

Examination of the 2D-IR spectra of **1** in heptane also showed that the intensities of both diagonal and off-diagonal peaks were observed to oscillate with *T*_w_ (Figure S6). These effects arise as a result of coherent processes involving simultaneous excitation of more than one ν_CO_ mode of **1** and have been observed previously.^21^ In the case of off-diagonal peaks, these are observed to oscillate at a frequency that matches the frequency separation of the involved, coupled, modes while diagonal peaks oscillate at frequencies that match the separations between the excited modes and other close-lying diagonal modes. Examples are shown in Figure S6 involving the diagonal peak corresponding to mode ν_1_ and the off-diagonal peak linking mode ν_1_ with ν_2_ (ν_2_ν_1_). Both were observed to oscillate at a frequency equivalent to 75 ± 1 cm^–1^, which corresponds to the frequency spacing of modes ν_1_ and ν_2_ (Figure 2). The dephasing time of the oscillations (2.5 ps) gives further insight into the time scale of local fluctuations of the molecule and solvent, which leads to loss of the coherence with time. In previous studies, it has been established that diagonal peak oscillations occur via the nonrephasing pathways that contribute to the 2D-IR signal while the off-diagonal peak oscillations occur via the rephasing pathways.^21^ These are observed simultaneously in our data because the pump–probe geometry collects both rephasing and nonrephasing terms.

## Discussion

One of the main benefits of applying 2D-IR spectroscopy to metallocarbonyl systems is the ability to gain detailed insight into the specific nature of the vibrational modes and potential energy surfaces of a molecule. In the case of **1**, the data show that the ν_CO_ modes are all mutually coupled. The implication is that excitation of any of the modes influences the frequencies of the remaining three such that the modes are delocalized to some degree across the four carbonyl ligands. This is also shown by the results of anharmonic DFT calculations.

The extent of the coupling and so delocalization does however vary between the modes. Although one of the primary factors in governing mode coupling strength is often the frequency separation of the modes, this does not account for the trend observed here. Alternatively, the observations may be rationalized by considering the nature of the atomic displacements involved in each mode (Figure 1). The most obvious case involves the pair of modes ν_2_ and ν_4_, which exhibit a negative coupling. Examination of the atomic displacements shows that these two modes that are almost entirely located on either the axial (ν_2_) or the equatorial (ν_4_) ligands, suggesting that this minimizes the coupling interaction. Considering the mode pairs that show positive couplings, very strong coupling occurs between modes ν_1_ and ν_2_ and between ν_3_ and ν_4_ (17 cm^–1^). Conversely, the coupling between modes in these two groups (i.e., ν_1_–ν_4_; ν_1_–ν_3_; ν_2_–ν_3_) is weaker, though not insignificant (5–8 cm^–1^). The origins of these effects are less clear. While the DFT results indicate that there is a slightly greater involvement of the axial ligand pair in mode ν_1_ and the equatorial ligand pair in mode ν_3_, there is sufficient delocalization to preclude assignment to solely axial or equatorial ligand modes and so no simple attribution can be made. This may also represent the fact that in *C*_2*v*_ complexes [M(L-L)(CO)_4_], the d_π_ orbitals split so that the orbital in the same plane as the L-L ligand (normally denoted as d_*x*2–*y*2_)^53,54^ makes a contribution to HOMO. The other two d_π_ orbitals (d_*xz*_ and d_*yz*_) make contributions to HOMO–1 and HOMO–2, which are similar energies.^45,53^ The latter two orbitals are, therefore, distinct from d_*x*2-*y*2_ and are involved in π-back bonding to the two mutually *trans* CO ligands.

When considering the vibrational dynamics of **1** in solution, it is noticeable that rapid energy transfer between the ν_CO_ modes is a key feature of the vibrational energy relaxation mechanism. This process has been widely observed in M(CO) species.^28,29^ The time scales for IVR between all modes is on the order of 5–10 ps, except for transfer between modes that show the greatest energy separation as described above. These observations are comparable with those on similar molecules in heptane solution.^28,34,55^ It is noticeable that the strength of the mode couplings discussed above does not clearly correlate with IVR time scales, though extraction of rates for a single mode-to-mode transfer is challenging in such a complicated system with overlapping peaks and multidirectional energy transfer processes to consider. The IVR process is widely accepted to be solvent mediated, with the low-frequency modes of the solvent acting as a bath, accepting or donating excess energy during the IVR transitions.^50^ This situation would dictate that the fastest energy transfer time would occur between modes that are separated by a frequency at which the low-frequency density of states of the solvent is at its peak.^52^ Typically, this peak falls between 50 and 90 cm^–1^ and pairs of modes with separations in this range do broadly show the faster IVR rates (Table 3), but this is by no means conclusive, perhaps again in part due to the complexity of the coupled manifold of ν_CO_ states involved in this system.

In the case of the overall vibrational relaxation time, the values of around 50 ps derived for the four carbonyl stretching modes of **1** in heptane are very short in comparison to measurements on similar systems, where values closer to 100 ps have been measured.^28,55^ One possibility is that the interactions between heptane and the ν_CO_ modes of **1** are stronger than usual. However, there is no evidence for this from the IVR time scales. The measured IVR times in the 5 ps range are consistent with the previous work,^55^ whereas strong solvent interactions would have been expected to lead to faster transfer rates. An alternative scenario is that the vibrational modes of **1** relax primarily via an intramolecular route. In particular, the large organic ligand of **1** will possess a significant number of low-frequency modes that might contribute to an increased number of channels for vibrational relaxation. This could explain the faster-than-usual relaxation times and the relative insensitivity of the relaxation time scale to the nature of the solvent, with values in the 30–50 ps range being measured across the four solvents studied. A related observation is that the lineshapes of the 2D-IR spectra showed little evidence of inhomogeneous broadening. This is consistent with previous studies carried out on metallocarbonyls in heptane solution^28,55^ and reinforces the view of a relatively limited degree of solvent–solute interactions taking place. In light of this, it is interesting to note that in activated complexes [Mn(N^∧^C) (solvent)(CO)_3_] where a solvent molecule replaces a photodissociated carbonyl ligand, the observed vibrational relaxation times are rather shorter. For example, in the imine analogues, *T*_1_ is 26.4 ± 0.8 ps.^15^ This is consistent with the binding of the solvent molecule increasing the solute–solvent interactions and bypassing the intramolecular relaxation mechanism, though changing the organic ligand will also affect the precise nature and quantity of low-frequency modes available to participate in relaxation.

The aim of the current study was to gain more detailed insight into the fundamental nature of the precatalyst **1** as a means to provide a basis from which the catalytic mechanism can be understood and so offer a potential route to optimizing the performance of derivatives. In this endeavor, the results provide the first data point rather than a conclusive answer. However, it has been demonstrated that changing the cyclomanganated ligand has an impact on reactivity^14,15,18^ and so this study establishes a baseline from which changes in molecular orbitals and bonding can be evaluated when different organic ligands are present. In addition, a first study of the interaction of the precatalyst with the solvent has been established, for comparison with later work. A more detailed investigation is now needed to establish whether these parameters link to reactivity, and if so, how. One potentially interesting avenue of enquiry would target coupling and energy transfer processes between CO and modes of the ppy ligand, though this lies beyond the scope of the current study.

It is especially encouraging to report the excellent agreement between DFT calculations and 2D-IR spectroscopy measurements in terms of predicting the mode descriptions, molecular structure, and couplings. As the latter arise from the complex interplay of the different normal mode potentials, this suggests that the predictive power of anharmonic DFT calculations will be a valuable tool in later studies, especially as a method to deconvolute the structure of reactive intermediates in catalytic cycles.

## Conclusions

In an effort to begin the process of understanding how the ground state potential energy surfaces and vibrational dynamics of a precatalyst species might influence reactivity, we have used 2D-IR spectroscopy in combination with anharmonic DFT calculations to study the molecule [Mn(ppy)(CO)_4_] in solution. Measurements of the coupling constants and angular relationships between the transition dipole moments of the ν_CO_ modes of **1** revealed the structure of **1** in solution. Determination of vibrational coupling parameters showed that all carbonyl stretching modes were coupled, indicating delocalization of the modes, though the absolute coupling strengths varied significantly depending on the precise nature of the atomic displacements, and so molecular orbitals, involved in each case. An investigation into the vibrational relaxation mechanism of **1** showed rapid IVR between the carbonyl stretching modes. A relative solvent independence of the overall vibrational relaxation time of **1** across a number of solvents of differing properties is suggestive of limited solvent–solute interactions.

Our study shows that the 2D-IR spectra of **1** show a rich and detailed pattern of off-diagonal peaks that are extremely well resolved such that we were able to clearly separate and identify contributions due to coupling and IVR. Such a nuanced spectral fingerprint would be useful in facilitating the evaluation of mixtures of species, for example, at points along the reaction coordinate as the nature of the catalytic species present altered. The ability to measure 2D-IR spectra on short time scales also opens up the interesting possibility of performing such analyses in real time. Of particular interest in subsequent studies will be how strongly the nature of the organic bidentate ligand modulates the measured parameters, given the clear role that this moiety plays in modulating reactivity.

## Supporting Information Available

Details of synthesis methods, additional ultrafast spectroscopy data, and analysis and results of DFT calculations (PDF)
