# Supplementary material for: Understanding the Vibrational Structure and Ultrafast Dynamics of the Metal Carbonyl Precatalyst [Mn(ppy)(CO)4]
Source: ACS Phys Chem Au. 2024 Jul 9;4(5):536–45. doi: 10.1021/acsphyschemau.4c00037 (PMC11428260; doi:10.1021/acsphyschemau.4c00037)
Supplement: Supplementary file 1 — pg4c00037_si_001.pdf [file pg4c00037_si_001.pdf]

Understanding the vibrational structure and ultrafast dynamics of the metal carbonyl pre-catalyst [Mn(ppy)(CO)$_4$]

Jonathan B. Eastwood,[1] Barbara Procacci,[1,2] Sabina Gurung, [1,2] Jason M. Lynam[1] and Neil T. Hunt[1,2] **

1) Department of Chemistry, University of York, York, YO10 5DD, UK

2) York Biomedical Research Institute, University of York, York, YO10 5DD, UK

Corresponding author email: neil.hunt@york.ac.uk ; jason.lynam@york.ac.uk

Table of Contents

Synthesis of **1**……………………………………..…………………………………..….………S2

Method for simulating 2D-IR spectrum from DFT results……………………………....….……...S3

Off-diagonal peak anisotropy analysis (Figure S1)…………………....….…………………....……S4

Results of DFT calculation (Table S1)..………………..…………………………………………...…S5

2D-IR spectra of [Mn(ppy)(CO)$_4$] in heptane solution at T_W from 250 fs to 100 ps (Figure S2)……...S6

IR pump-probe spectroscopy results and vibrational relaxation dynamics and of **1** in different solvents (Figure S3)………………...…………………………………………………………...….……..S7

Energy transfer (IVR) kinetics analysis (Figure S4)………………..……………….………………S8

Anisotropy plots determined by IR PP spectroscopy (Figure S5)…………...S10

Coherent oscillation involving modes ν_1 and ν_2 (Figure S6)…………………....…………...……S11

References …………………………………...………………………………………………....S11

General experimental

Synthesis of BnMn(CO)$_5$

To a Schlenk tube under nitrogen was added mercury (12 ml). Sodium metal (1.18 g, 42.8 mmol, 4 eq.) was added in small pieces with high stirring to allow dissolution. In a separate Schlenk tube under nitrogen was added $Mn_2(CO)_{10}$ (4.16 g, 10.7 mmol, 1 eq.), followed by dry deoxygenated THF (80 ml). The THF solution was then transferred by cannula to the sodium amalgam, which was stirred for a further 3 hours. Benzyl chloride (2.47 ml, 2.71 g, 21.4 mmol, 2 eq.) was added to a separate Schlenk tube under nitrogen and placed in an ice bath. The Schlenk tube was put under vacuum with stirring for 30 seconds, backfilled with nitrogen. At room temperature, the THF solution was transferred by cannula filtration to the benzyl chloride and stirred for 20 hours. The solution was thereafter filtered through a bed of Celite® and washed with Et_2O. The crude material was loaded onto silica gel and purified by flash column chromatography (petrol). Benzyl chloride impurities was removed under reduced pressure at 35 °C to yield a pale green crystalline solid (4.11 g, 67%). Mp 114–115 °C (lit[1] 40 °C); R_f 0.33 (petrol); ^{1}H NMR (500 MHz, $CDCl_3$, δ): 7.24– 7.12 (m, 4H), 7.05–6.94 (m, 1H); ^{13}C NMR (126 MHz, $CDCl_3$, δ): 212.4, 210.0, 151.9, 128.8, 126.0, 123.6, 11.3; LIFDI-MS m/z (ion, %) 286 $[M]^+$; IR (CH_2Cl_2, solution, cm^{-1}): 2940, 2874, 2857, 2107, 2011, 1990, 1598, 1489, 1124. The analytical data obtained were in accordance with the literature.[1]

Synthesis of Mn(ppy)(CO)$_4$

To a Schlenk tube under nitrogen was added MnBn(CO)$_5$ (2.50 g, 9.15 mmol, 1 eq.), 2-phenylpyridine (1.33 ml, 1.45 g, 9.15 mmol, 1 eq.) and dry deoxygenated hexane (60 ml). The reaction mixture was excluded from light and heated to reflux for 6 hours, until completion of the reaction given from monitoring by IR. When the reaction was complete, the reaction mixture was allowed to cool to room temperature and was filtered through cotton wool. CH_2Cl_2 was used to rinse the flask and cotton wool, before the solvent was removed under reduced pressure. The crude material was purified by flash column chromatography to afford the product.Purification by flash column chromatography (petrol/CH_2Cl_2, 8:2, v/v) to afford a yellow crystalline solid (2.37 g, 81%). Mp 114–115 °C (lit[1] 114 °C); R_f 0.26 (petrol/CH_2Cl_2, 8:2, v/v); ^{1}H NMR (500 MHz, $CDCl_3$, δ): 8.00 (d, J = 7.5 Hz, 1H), 7.89 (d, J = 8.0 Hz, 1H), 7.84–7.74 (m, 2H), 7.29 (dd, J = 7.5, 7.5 Hz, 1H), 7.19 (dd, J = 7.5, 7.5 Hz, 1H), 7.12 (dd, J = 6.5, 6.5 Hz, 1H); ^{13}C NMR (126 MHz, $CDCl_3$, δ): 220.2, 214.3, 214.1, 174.9, 166.5, 154.0, 146.3, 141.8, 137.9, 130.4, 124.2, 124.1, 122.5, 119.4; LIFDI-MS m/z (ion, %): 321 ($[M]^+$,

100); IR (n-Bu_2O, solution, cm^{-1}): 2075, 1992, 1978, 1937, 1604, 1579, 1479. The analytical data obtained were in accordance with the literature.[1]

Simulating 2D IR spectra

Each individual peak was simulated using the 2D-Gaussian equation shown in (equation 1) and summed to generate full spectra.

$$f(x,y) = A \exp\left[-\frac{1}{2(1-C_{2D}{}^2)}\left(\left(\frac{x-x_0}{\sigma_x}\right)^2 + \left(\frac{y-y_0}{\sigma_y}\right)^2 - \frac{2C_{2D}(y-x_0)(X-y_0)}{\sigma_x\sigma_y}\right)\right] \qquad (1)$$

Where:

f(x,y) = signal amplitude at pump wavenumber, probe wavenumber of (x,y)

x = probe wavenumber

y = pump wavenumber

A = amplitude of peak

C_{2D} =cross correlation parameter

x_0 = central wavenumber of peak along probe axis

y_0 = central wavenumber of peak along probe axis

σ_x = standard deviation along probe axis

σ_y = standard deviation along pump axis

$$\sigma_x = \frac{\mathrm{FWHM}}{2\sqrt{2\ln(2)}}$$

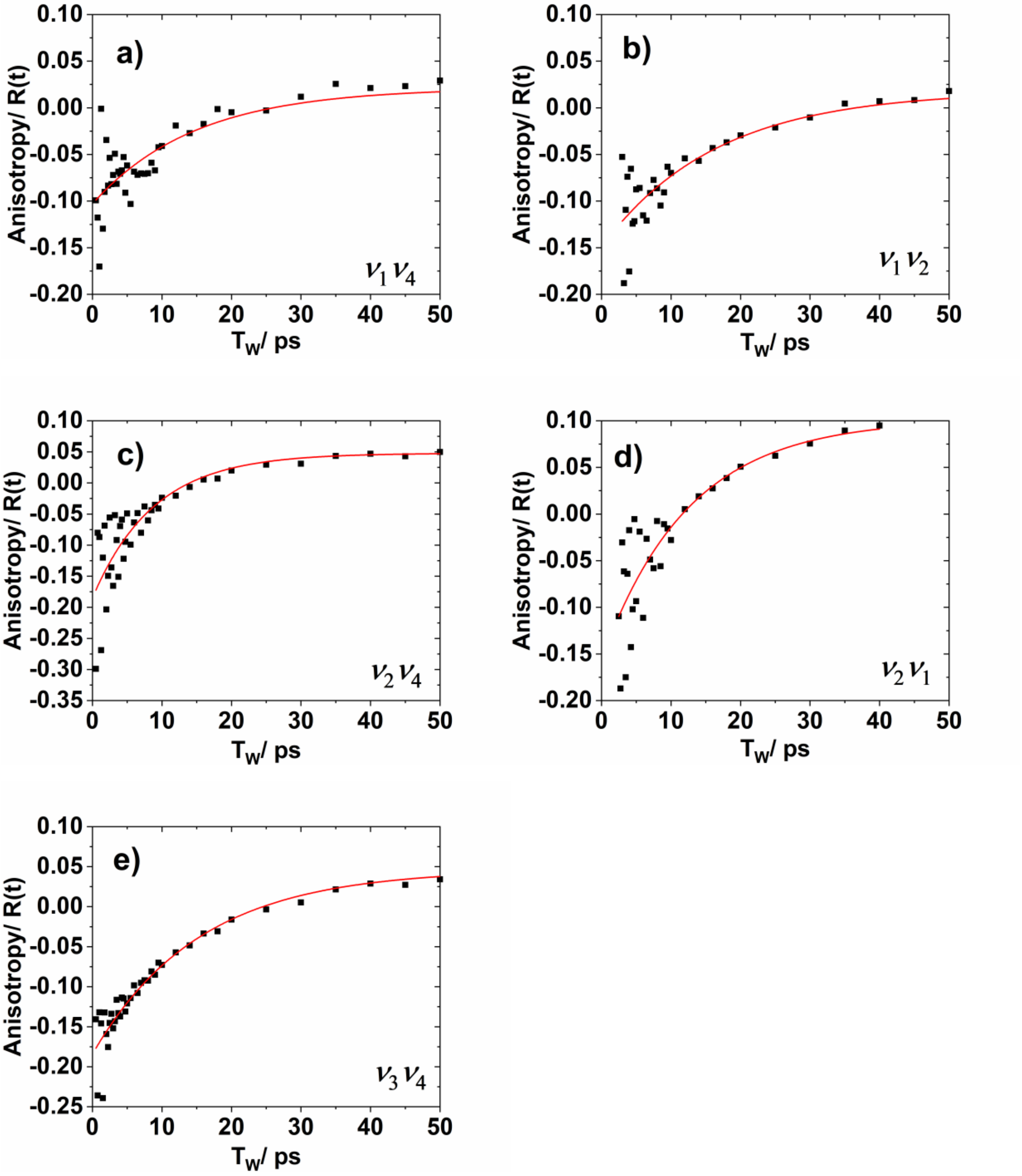

Figure S1. *Off-diagonal peak anisotropy analysis (a-e) for determination of angle between transition dipole moments (Table 1 in the paper). The black squares are experimental data points and the red line shows the best exponential fit to the points. A value of -0.2 at* $T_w = 0$ *ps indicates a TDM angle of 90°. Figure labels indicate specific cross peak in the format* $\nu_{pump}\nu_{probe}$.

Table S1: *Results of DFT calculations including harmonic frequencies, anharmonic frequencies and inter-mode couplings for the MC≡O vibrational modes in* ***1****. Coordinates of* ***1*** *are quoted below the table.*

Mode	Harmonic frequency / cm^{-1}	Relative Intensity / cm^{-1}	Anharmonic Frequency / cm^{-1}	Anharmonicity / cm^{-1}	ν_1 coupling / cm^{-1}	ν_2 coupling / cm^{-1}	ν_3 coupling / cm^{-1}	ν_4 coupling/ cm^{-1}
ν_1	2078.7	0.17	2050.4	7.4		18.1	9.1	5.8
ν_2	2000.0	1.00	1972.7	11.6	18.1		5.5	-0.6
ν_3	1995.5	0.42	1967.6	6.6	9.1	5.5		15.9
ν_4	1965.5	0.58	1938.6	13.2	5.8	-0.6	15.9	

Energy and Coordinates of ***1***

Energy = -2082.547615 Hartrees

Mn	0.77909200	-0.83168500	0.00000000
C	0.72999400	-2.65423300	0.00000000
O	0.73405600	-3.82151100	0.00000000
N	-1.30076800	-0.59626300	0.00000000
C	-4.06139300	-0.09406900	0.00000000
C	-3.57640100	-1.41442600	0.00000000
C	-2.19153400	-1.61446100	0.00000000
C	-1.75434800	0.69955700	0.00000000
C	-3.14497100	0.96175400	0.00000000
H	-5.14535600	0.10947300	0.00000000
H	-4.25357500	-2.28315100	0.00000000
H	-1.77237900	-2.63262700	0.00000000
H	-3.50123200	2.00285500	0.00000000
C	0.76138300	-0.65094600	1.83354700
C	0.76138300	-0.65094600	-1.83354700
O	0.76138300	-0.50432600	2.98407900
O	0.76138300	-0.50432600	-2.98407900
C	2.57550400	-0.80403900	0.00000000
O	3.74155500	-0.77124300	0.00000000
C	0.63956900	1.23465700	0.00000000
C	0.08358200	4.03574300	0.00000000
C	1.41235800	3.56786600	0.00000000
C	1.68143900	2.18579100	0.00000000
C	-0.70129000	1.72239300	0.00000000
C	-0.97094100	3.11386200	0.00000000
H	-0.12819700	5.11829800	0.00000000
H	2.25005300	4.28752900	0.00000000
H	2.73514100	1.85732500	0.00000000
H	-2.00939600	3.48648800	0.00000000

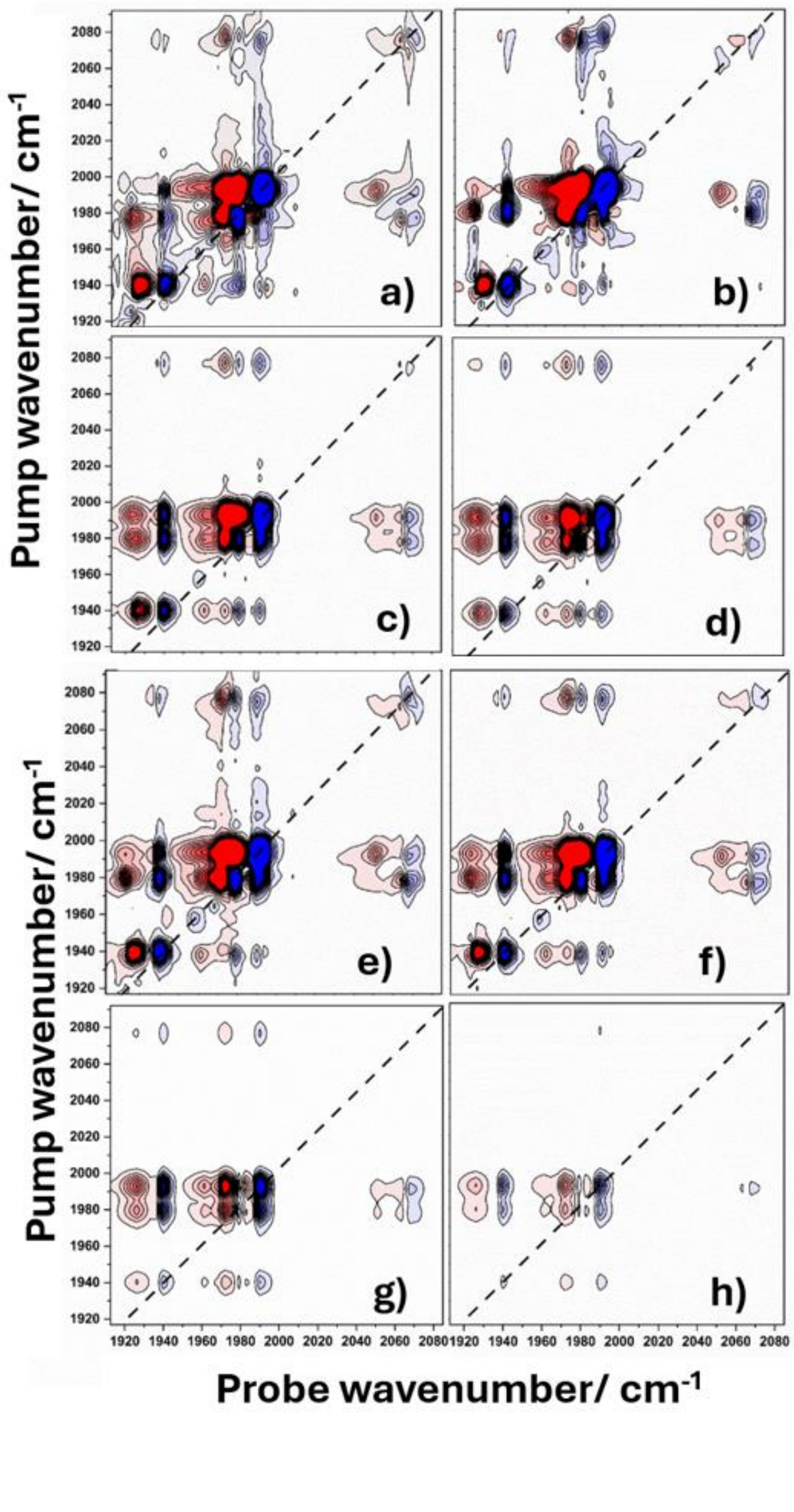

Figure S2. *2D IR spectra of [Mn(ppy)(CO)$_4$] in heptane solution with ZZZZ pump polarization at T_W values of 250 fs (a), 1 ps (b), 2.5 ps (c), 5 ps (d), 10 ps (e), 20 ps (f), 50 ps (g), and 100 ps (h). The spectra were scaled to the same intensity axis, the scale runs from blue (negative) to red (positive).*

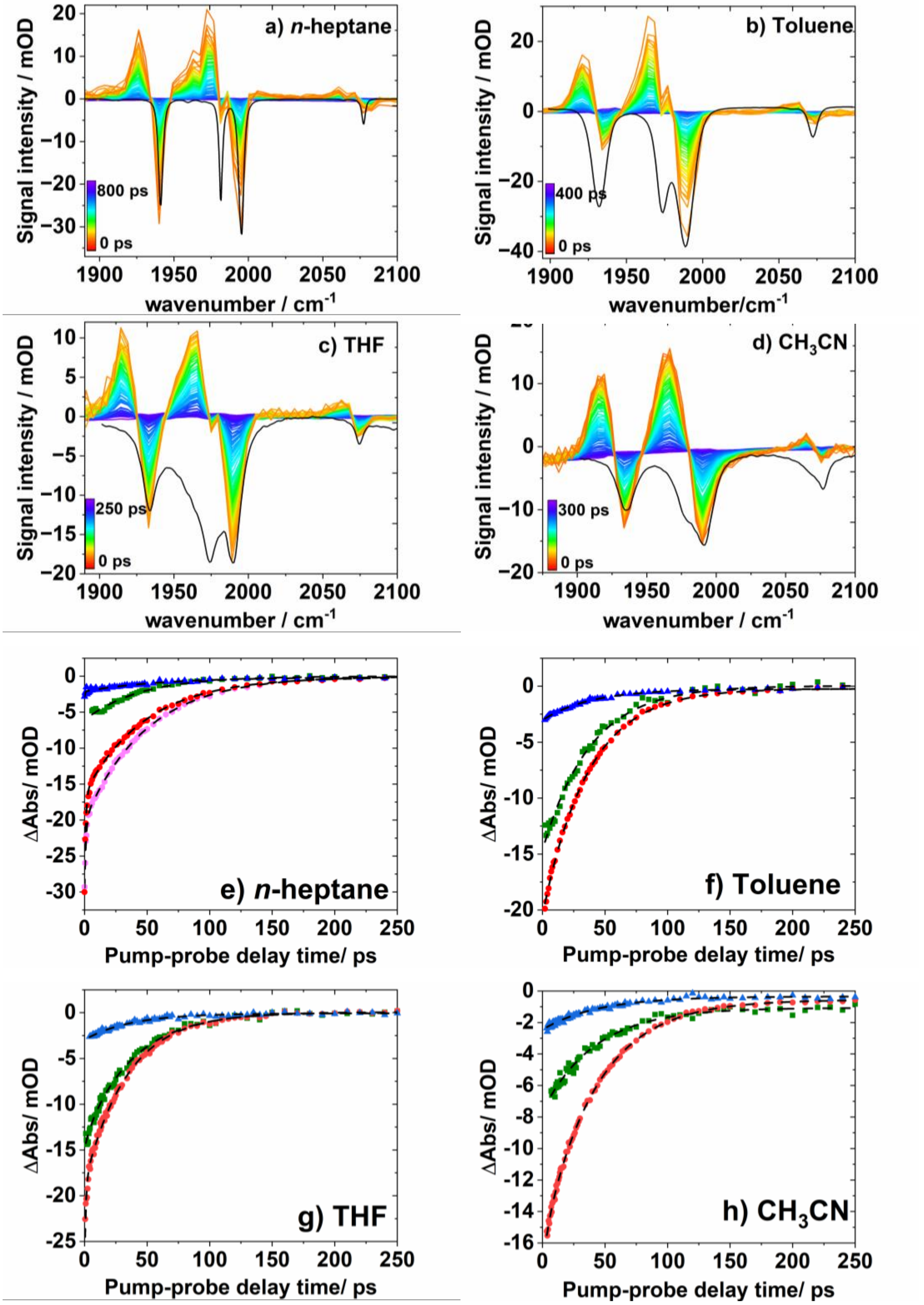

Figure S3. *a-d) Pump-probe IR spectra of **1** in (a) n-heptane, (b) toluene, (c) THF, (d) CH_3CN solution. The black traces represent the respective superimposed ground state FTIR spectrum. e-h) Vibrational relaxation dynamics of the ν_{CO} modes of **1**. Graphs show change in amplitude of the v=0-1 transitions with pump-probe delay time, obtained using magic angle polarization in (e) n-heptane, (f) toluene, (g) THF, (h) CH_3CN solution. In each graph, blue triangles show data points obtained for the ν_1 mode, red circles (ν_2), magenta hexagons (ν_3) and green squares (ν_4), the black lines are the best fits through the points using an exponential function. (Table 2 in the main text)*

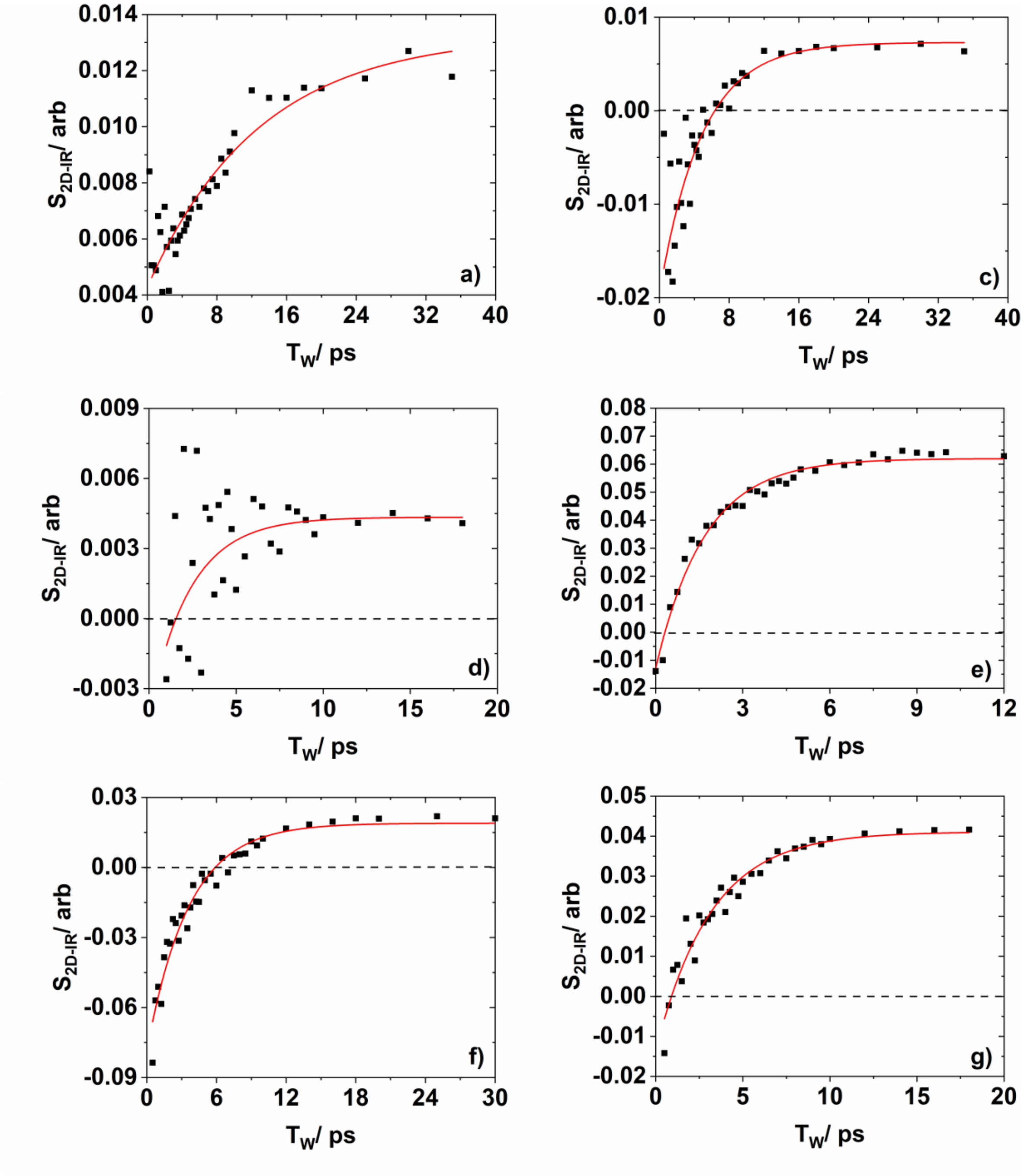

a)
b)
c)
d)
e)
f)
g)
S2D-IR/ arb
TW/ ps

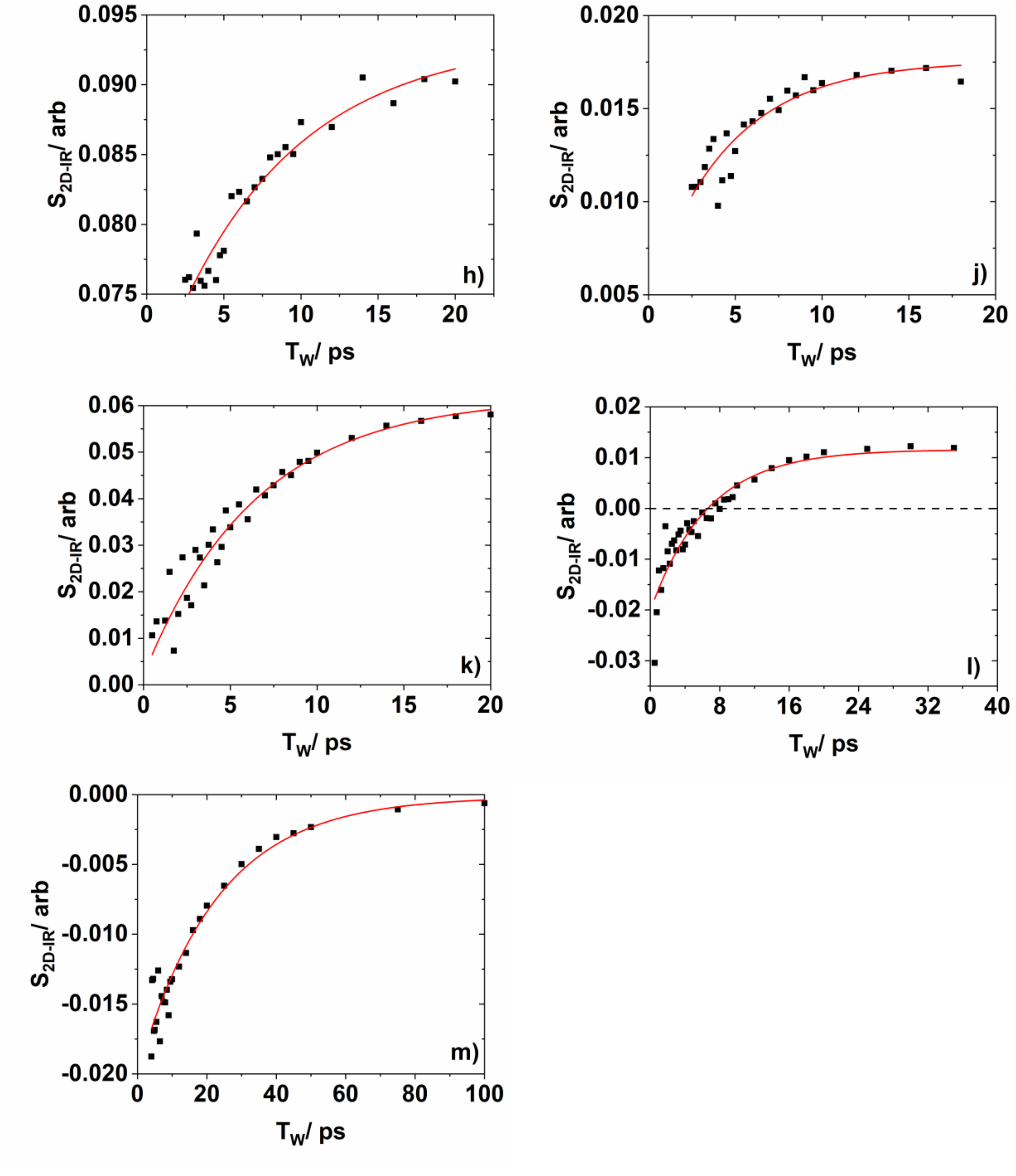

Figure S4. *Energy transfer peak (a-m) kinetic analysis; the black squares represent the experimental data and the red lines are the best fit to them (Table 3 in the paper). Black dotted line added at* $S_{2D\text{-}IR}$ *= 0. Some kinetic traces start below or above 0 due to peak overlap and cancellation.*

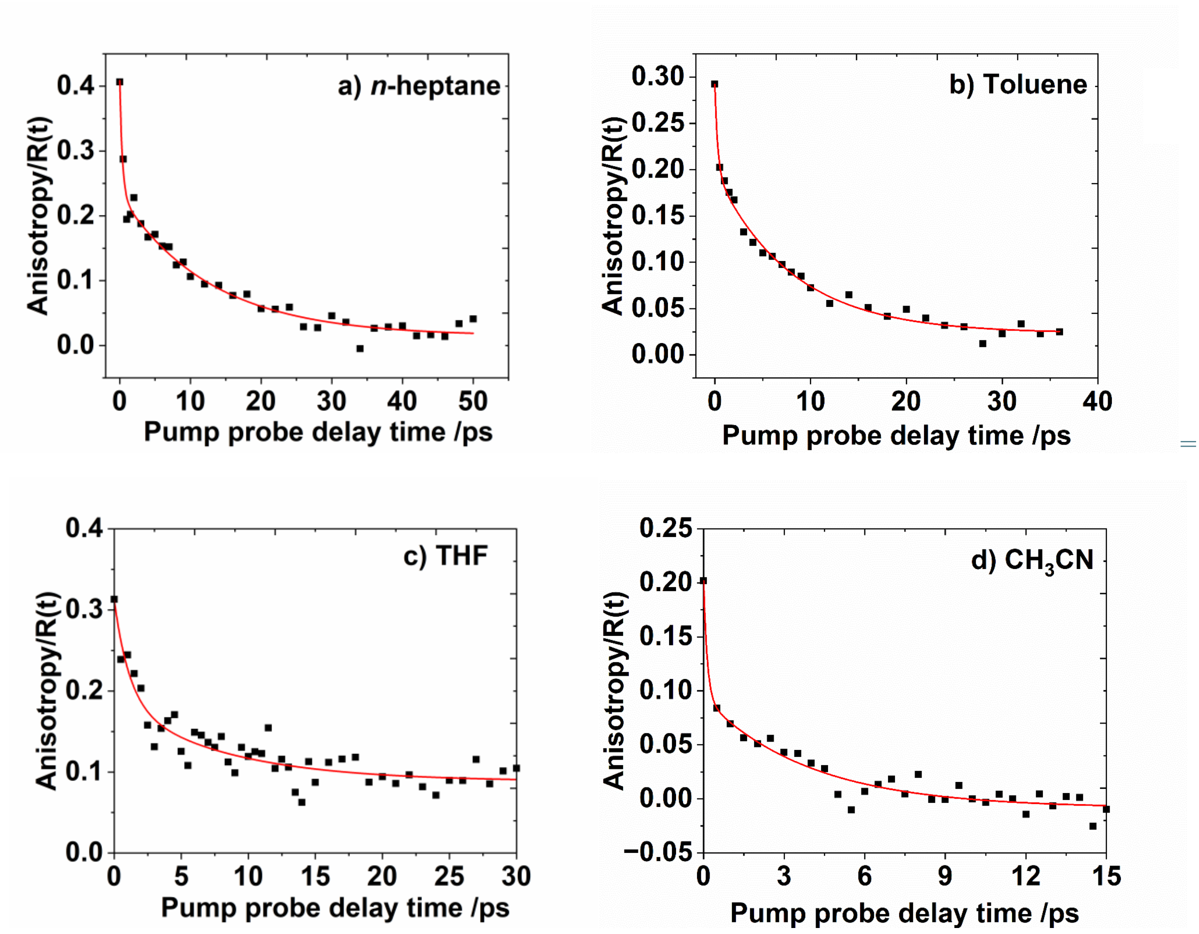

Figure S5: *Anisotropy decays determined from IR pump-probe spectroscopy of* ν_2 *of* ***1*** *in (a) n-heptane, (b) toluene, (c) THF and (d)* CH_3CN *solution. The black squares represent the experimental data and the red lines are the best fit using a bi-exponential decay function. Anisotropy calculated by the following equation:* $Anisotropy = \frac{S_{ZZZZ} - S_{ZZYY}}{S_{ZZZZ} + 2 \times S_{ZZYY}}$.

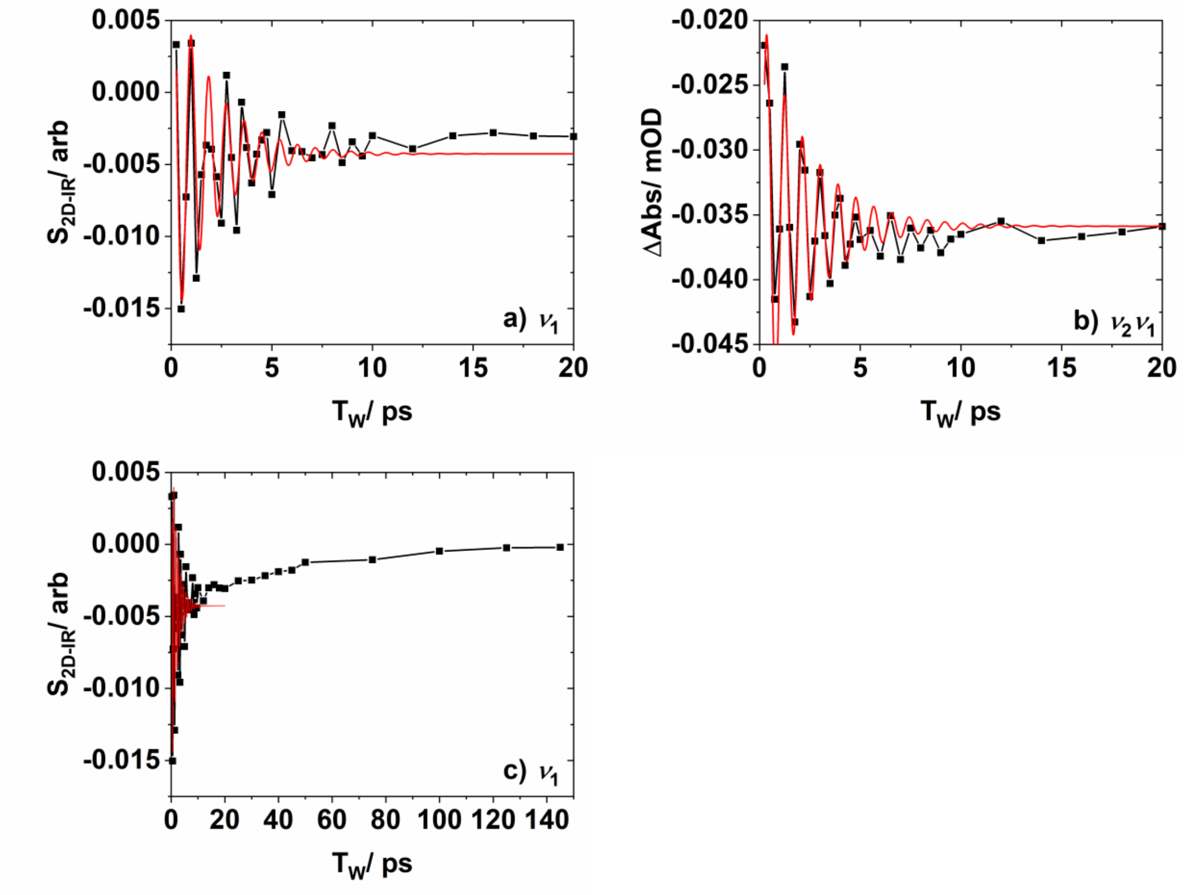

Figure S6. *Coherent oscillations observed for (a) the diagonal peak corresponding to mode* ν_1*, (b) the off-diagonal peak linking modes* ν_2 *and* ν_1 *(*$\nu_2\nu_1$*) of* ***1*** *and (c) kinetics for the diagonal peak corresponding to mode* ν_1 *at* T_W *values of up to 145 ps. Beat frequencies and decay times were determined by fitting monoexponentially damped sine functions red lines) to the experimental data (black squares). Oscillation frequencies were determined to be 74 cm*$^{-1}$ *(a) and 76 cm*$^{-1}$ *(b) which correspond to the frequency difference between mode* ν_1 *and* ν_2*. Decay constants were determined to be 2.1 ± 0.5 ps (a) and 2.3 ± 0.4 ps (b).*

References

1) Jonathan S. Ward, Jason M. Lynam, James W. B. Moir, David E. Sanin, Adrian P. Mountford and Ian J. S. Fairlamb, Dalton Trans., 2012, 41, 10514-10517.
